# Prevalence and Trend of Overweight and Obesity among Schoolchildren in Ahvaz, Southwest of Iran

**DOI:** 10.5539/gjhs.v6n2p35

**Published:** 2013-11-26

**Authors:** Hamed Tabesh, Sayyed Mahdi Hosseiny, Farshid Kompani, Azadeh Saki, Maliheh Saeed Firoozabadi, Roghayeh Chenary, Mahta Mehrabian Fard

**Affiliations:** 1 Department of Biostatistics and Epidemiology, School of Health, Ahvaz Jundishapur University of Medical Sciences, Ahvaz, Iran; 2 Golestan Hospital, Ahvaz Jundishapur University of Medical Sciences, Ahvaz, Iran; 3 Golestan Hospital, Ahvaz Jundishapur University of Medical Sciences, Ahvaz, Iran; 4 Department of Health, Bushehr University of Medical Sciences, Bushehr, Iran; 5 Health Center, Ahvaz Jundishapur University of Medical Sciences, Ahvaz, Iran

**Keywords:** Body Mass Index (BMI), overweight, obesity, prevalence, schoolchildren

## Abstract

**Introduction:**

Obesity is an important risk factor for some chronic diseases. Since the effect of obesity is long-standing, monitoring childhood obesity should be the first step in the health policy for interventions regarding early prevention of chronic diseases. In this study we aim to determine the prevalence of overweight and obesity among school children in the city of Ahvaz.

**Methods:**

A cross-sectional survey was designed. A sample of 5811 children, 2904 (49.97%) boys and 2907 (50.03%) girls, was selected and their heights and weights were measured in 2012-2013 academic year. Measurements of height and weight were made by using calibrated equipment and according to standardized protocol with the children having light clothes and without wearing shoes. The adjusted odds ratio of obesity and overweight for age and sex were calculated from multiple logistic regression model.

**Results:**

A total 685 (23.6%) of boys and 561 (19.3%) of girls were overweight. and 190(6.05%) of boys and 130 (4.5%) of girls were obese. The proportion of overweight and obese boys was significantly higher than that of girls (p<0.001). Logistic regression showed significant increase in the likelihood of being overweight with the increasing age OR=1.50, C.I.95%: (1.43, 1.57).

**Conclusion:**

The prevalence of overweight and obesity increased markedly with age. This shows the importance of early prevention by doing interventions and training since the first year of primary school.

## 1. Introduction

Over time, definition of obesity and overweight has changed; it can be defined as a surplus of body fat (Christoffel et al., 2012). Body mass index is a useful index for weight relative to stature. High Body Mass Index (BMI) is a public health concern especially among children adolescents. This is because children with high BMI often become obese adults in future. Some of the important consequences of high BMI are hypertension and hyperlipidemia and high blood pressure (Ogden et al., 2010). Also, they catch with chronic disease, such as heart disease, cancers, diabetes (Armstrong et al., 2008; Christoffel et al., 2012; Dehghan et al., 2005; Ogden et al., 2010; Zaghami et al., 2013). Factors that influence childhood obesity begin from early stage in life. These factors have been studied in different domains, such as genetics, psychology, and epidemiology, and age range; prenatal, first 1-2 years of life, and the preschool/early primary school years (Christoffel et al., 2012). According to experimental and observational studies, regular exercise may have a positive effect in reducing the amount of body fat in children. In brief, both diet and physical activity have an impact on reducing obesity, This shows the important role of parents to control diet and physical activity in their children as a means to control the weight of their offspring (Dehghan et al., 2005).

Health status has largely improved in children and adolescents as a result of control of communicable diseases and declines in nutrient deficiencies of the past (Payandeh et al., 2013a; Payandeh et al., 2013b; Saki et al., 2013; Saki et al., 2010); but westernization and changing in lifestyle made them prone to non-communicable diseases such as obesity or overweight later in life (De Onis et al., 2010). In 1998, the World Health Organization reported that Iran is one of the seven countries with the highest prevalence of childhood obesity. For instance, the prevalence of BMI between 85th and 95th percentile in girls was significantly higher than that in boys (Dehghan et al., 2005).

The prevalence of childhood obesity and overweight in Iran has been investigated in some studies from different regions. Taheri and collogues performed a study on 6093 students on 2009 with the aim of determining the prevalence of overweight and obesity in 7 to 18 year old children in Birjand in Iran. Findings illustrated that the rates of overweight and obesity were 4.8% and 1.8% respectively. The prevalence of overweight was different by age from 1.6% to 9.1% in girls and 0.5% to 7.8% in boys. Also, the rates of obesity were 0.8% to 2.5% in girls and 0.5 to 3.7 in boys (Taheri & Kazemi, 2009). Mirmohammadi and collogues designed a study on 2011 in Yazd, center of Iran, on 30092 Iranian children aged 7-18 years in six ethnic groups, titled prevalence of Overweight and Obesity among Iranian school children in Different Ethnicities. The results showed that the prevalence of overweight and obesity among Iranian children was 9.27% and 3.22% respectively. The frequency of overweight and obesity was higher in boys. Also this study illustrated a significant difference in BMI among different ethnic groups. Main reasons for variation of overweight and obesity prevalence may be due Geographic, cultural and also ethnic differences. It also may be due to their different lifestyle and different nutrition in different ethnic groups (Mirmohammadi et al., 2011).

The present study conducted to determine BMI percentiles and also the prevalence of overweight and obesity of school children in Ahvaz, the center of Khuzestan province of Iran. It is in the south-west of Iran, with a hot and humid weather with having very hot and wet weather. This city is one of the most developed and also industrial cities of the country. The World Health Organization ranked Ahvaz as the most air polluted city of the world in 2011. The second objective of this study is to evaluate the prevalence of obesity and the influence of age on obesity among school children in Ahvaz.

## 2. Subjects and Methods

We concluded a cross-sectional study on the prevalence of obesity and overweight among school children in Ahvaz city, south-west of Iran in 2012. Healthy 7-11 year old boys and girls were selected from 4 districts of Ahvaz. Multistage random sampling technique was used. At the first stage, stratified sampling was conducted based on four districts, at the second stage, two schools were selected at each stratum by simple random sampling. Whole students of 8 selected schools contributed to the study and anthropometric parameters were obtained from 7-11 year old students. The WHO child growth standards (2007) were used for analysis. Measurements of weight were obtained by calibrated equipment according to a standardized protocol, with the students having light clothes and without wearing shoes. The study population consisted of 5811 children aged 7-11 years which comprised about 10% of all primary school students selected from 4 districts of Ahvaz.

Data were collected from the students according to a check-list. After that, data were coded and analyzed by SPSS software and the age and sex specific prevalence of obesity and over weigh and confidence intervals were calculated. In addition multiple logistic regression was used to find the odds ratios of age and sex for obesity and overweight.

## 3. Results

This cross-sectional study was performed among 2904 boys (49.97%) and 2907 girls (50.02) living in Ahvaz city in the south-west of Iran. Table 1 shows age-related prevalence of obesity and overweight among primary school children in Ahvaz. Of all participants, 685 boys and 561 girls were overweight and obese, and the difference between them was significant (p value <0.0001). In this case, the prevalence of obesity was higher in boys (23.6%) than girls (19.3%). The number of 190 boys and 130 girls were obese and difference between them was significant (p=0.001). This means that the prevalence of obesity was higher in boys (6.5%) than girls (4.5%).

**Table 1:** Age-Related Prevalence of Obesity among school children in Ahvaz by sex, 2013

Age (years)	Boys			Girls			P-value for sex	
	N	Overweight n (%)	Obese n (%)	N	Overweight n (%)	Obese n (%)	Overweight	Obese
7	604	55 (9.1)	9 (1.5)	612	44 (7.2)	11 (1.8)	0.222	0.674
8	616	95 (15.4)	20 (3.2)	609	92 (15.1)	3 (0.5)	0.878	< 0.001
9	598	170 (28.4)	61 (10.2)	586	114 (19.4)	37 (6.3)	< 0.001	0.015
10	548	170 (30.9)	56 (10.2)	550	158 (28.3)	46 (8.2)	0.406	0.290
11	525	195 (36.4)	44 (8.2)	521	153 (28.4)	33 (6.1)	0.008	0.205
All	2904	685 (23.6)	190 (6.5)	2907	561 (19.3)	130 (4.5)	< 0.001	0.001

The percentiles of BMI by age are presented in Table 2 separately for boys and girls. The median of BMI among boys at the first year of primary school is 15.03 and then reached to 18.26 at the last year and among girls these are 14.54 and 18.12, respectively. However the median of BMI is similar between boys and girls. The 3rd percentiles of BMI among girls are lower than boys and the 97th percentiles of BMI among girls are higher than boys at all ages, so the variation of BMI among girls is more than boys.

**Table 2:** Presents smoothed percentiles of BMI values for thin, median, overweight and obese infants at specified ages

Age	BMI percentile									
	Boys					Girls				
	P3	P50	P85	P97	N	P3	P50	P85	P97	N
7	12.42	15.03	16.52	18.31	604	12.10	14.54	16.38	18.74	612
8	13.01	15.54	17.56	19.51	616	12.21	15.80	17.84	19.51	609
9	13.23	16.39	19.49	21.63	598	12.77	16.22	18.94	22.33	589
10	13.25	16.76	20.08	22.97	551	13.32	17.33	21.00	23.63	559
11	14.88	18.26	21.27	23.49	535	14.49	18.12	21.43	25.09	539

**Figure 1:**
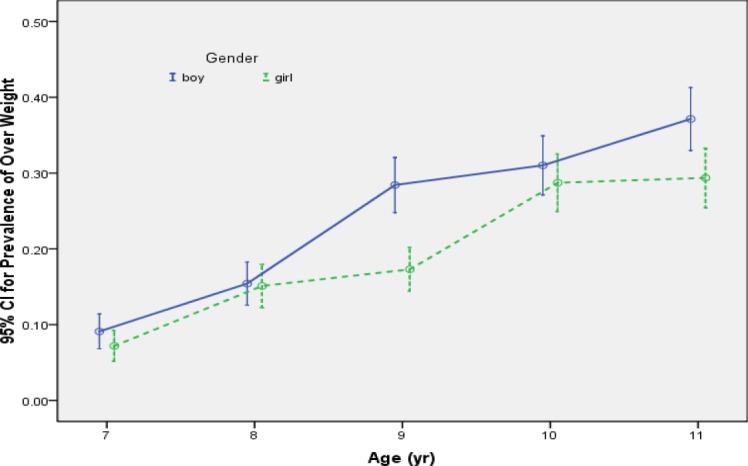
Prevalence of overweight by age and sex

Figure 1 shows the 95% confidence intervals for prevalence of overweight versus age for boys and girls. As shown in this figure the prevalence of overweight increased rapidly by age and the prevalence of overweight among girls is lower than boys.

**Figure 2:**
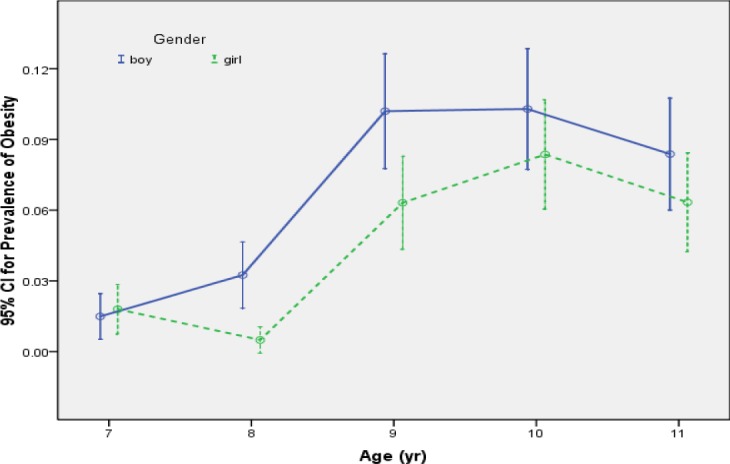
Prevalence of obesity by age and sex

The 95% confidence intervals for prevalence of obesity versus age for boys and girls show in Figure 2. As shown in this figure the prevalence of obesity in age 9 considerably increased rather than age 8 in both sex and then decreased gradually.

Multiple logistic regressions were used to estimating the odds ratio of sex and age for obesity and overweight. The results are shown in table 3. The odds ratios of overweight and obesity among boys are 1.34 and 1.50, respectively. And the odds of overweight increased 1.50 times yearly. Since the prevalence of obesity between 9-12 years old children is considerably higher than 7-8 years old we recode the age of children in to two groups : equal or less than 8 yrs / greater than 8 yrs.

**Table 3:** The adjusted OR of overweight and obesity from multiple logistic regressions

Response variables	Predictors	B	S.E.	P-Value	Adjusted OR	95.0% C.I. for OR	
						Lower	Upper
Overweight	Gender	.291	.066	.000	1.338	1.176	1.523
	Age	.405	.024	.000	1.499	1.428	1.572
Obesity	Gender	.404	.118	.001	1.498	1.188	1.888
	Age (>8/<=8)	1.625	.166	.000	5.080	3.667	7.036

## 4. Discussion

Prevalence of overweight among boys and girls showed a steady increase with age from 7 to 11 years. But the prevalence of obesity among 7-8 Year old school children was similar. There was a considerable increase in the prevalence of obesity among 9 year olds which then showed a slight decrease. The overweight, obesity, and BMI percentiles were higher in boys than girls, and previous studies were supported this finding (^2,13^; Taheri and Kazemi, 2009). The age and sex related centiles of BMI for the school children of Ahvaz offer an opportunity to monitor an individual’ s degree of fatness and have the practicality as a simple technology for preventing overweight and obesity related problems. A comparison of our data with measurements taken in other studies in different regions of Iran (Ayatollahi and Mostajabi, 2007; Mirmohammadi et al., 2011; Tabesh et al., 2010; Taheri and Kazemi, 2009) shows a considerable difference on prevalence of overweight and obesity among schoolchildren for both boys and girls in Ahvaz. This may be attributed to limited outdoor activities because of climatic condition, air pollution, social conditions, and cultural preferences among some sub-communities and social classes. Other factors that may be involved are frequent feeding habit from birth and westernization of feeding habits with the frequent use of fast foods among Iranian children (Ayatollahi and Mostajabi, 2007; Saki et al., 2013). Changing dietary habits and physical activity patterns because of rapid urbanization and transport facilities and untargeted governmental subsidy for energy-dense foods (e.g. fat and sugar) are identified as the reasons for the great prevalence of overweight and obesity for schoolchildren in Ahvaz.

Although the prevalence of overweight and obesity in developed countries is about double that in developing countries (11.7% and 6.1%, respectively), the vast majority of affected children (35 million) live in developing countries. In addition, the relative Rate of increase in the past 2 decades has been higher in developing countries (+65%) than in developed countries (+48%). The study was conducted by Cattaneo and collogues to review overweight and obesity in infants and pre-school children in the European Union in 2006, all values were higher in girls than boys (Cattaneo et al., 2010; De Onis et al., 2010). Also, Armstrong and collogues reviewed Obesity and overweight in South African primary school children-the health of nation in a sample of 10,195 children aged 13-6 and it was found that the prevalence rates of overweight and obesity in girls were higher than boys, However, in our study, these values were higher in boys than girls that is according to another study by Armstrong et al, in 2008. In Khader study about the prevalence of overweight among school children 6-12 years old, it has been shown that overweight were higher in girls than boys, However, the prevalence of obesity was higher in boys than girls, which is similar to a recent finding (Khader et al., 2009). According to another study in India the prevalence of overweight was higher in girls than boys (Sidhu et al., 2006). The main reasons of this difference may be due to Geographical and cultural differences. In explanation of cultural differences it can be said that gender preferences may play an important role so that there is a preference for sons over daughters. This may result in a higher food intake and a lower physical activity by boys with a consequent higher prevalence of obesity among boys than girls. According to a study by Badawi and colleagues in Egypt in 2012 the prevalence of overweight was 17.7% and that of obesity was 13.5%. The rate of obesity was high in children aged 7-8 but by increasing in their age this rate will be decreased, while the prevalence of overweight increased by the increases in age (Badawi et al., 2013).

According to Kuokawa study in Japan (an Asian developing country) the mean prevalence rate of overweight and obese children were 19.5% and 4.1% for boys in 6thPS (primary school) 13.6% and 2.2% for girls in 6thPS, 13.6% and 3.0% for boys in 3rdJHS (junior high school student), and 12.2% and 1.9% for girls in 3rdJHS, respectively (Kurokawa & Satoh, 2011). In a study by Dekkaki in Morocco on primary school chidren of low socioeconomic status, the prevalence of overweight and obesity was 8.7%. overweight was 5.1% and obesity was 3.6% (Cherkaoui Dekkaki et al., 2011). In another study by Scarlett and colleagues on the prevalence of overweight and obesity among children six to ten years of age in in Jamaica in 2013 the prevalence of overweight and obesity was 10.6% and 7.1%, respectively. The prevalence of overweight and obesity increased with age significantly. In that study the prevalence of overweight and obesity was higher among girls than boys significantly (Blake-Scarlett et al.). This finding is similar to our findings. It may be due to urban children in private schools have higher prevalence than rural school children. So, health promotion interventions are required to control this problem. Kaur and collogues conducted a study titled prevalence of overweight and obesity in school children in Delhi in 2008. This study documented that the prevalence of overweight and obesity for all the age groups was higher in children from average- income families as compared to those of low-income group (Kaur & Kapil, 2008). According to a study in India in 2011 by Patnaik at Orissa, on 468 schoolchildren, prevalence of overweight and obesity in schoolchildren of 5-15 years was 28.63% (overweight – 14.1% and obesity – 14.53%). Maximum prevalence 36.54% was found in children aged 5-10 years and 33.65% in boys (Patnaik et al., 2011).

The results of our study in terms of the prevalence of obesity are similar to the study in Egypt with similar findings. The study from India reported higher prevalence of obesity and overweight than our study, all the other studies from other regions of Iran and also the other countries reported lower prevalence of obesity and over weight in children Compared to to our study. As was mentioned the prevalence of overweight and obesity among schoolchildren in Ahvaz is higher than other regions in Iran (Ayatollahi & Mostajabi, 2007; Mirmohammadi et al., 2011; Taheri & Kazemi, 2009). The odds ratio of obesity and overweight among schoolchildren increased markedly with age. This necessitates emergency measures to be taken in order to prevent overweight and obesity in schoolchildren strongly recommended.

This study is the first findings about the prevalence of obesity and overweight in school children in Ahvaz. We recommend a prospective study for determining the prevalence secular trend and risk factors of overweight and obesity in this population. It is clear that a cross-sectional study, as we do, could not be very validate about trend.
